# Author Correction: Chemical trends of deep levels in van der Waals semiconductors

**DOI:** 10.1038/s41467-020-20151-x

**Published:** 2020-11-26

**Authors:** Penghong Ci, Xuezeng Tian, Jun Kang, Anthony Salazar, Kazutaka Eriguchi, Sorren Warkander, Kechao Tang, Jiaman Liu, Yabin Chen, Sefaattin Tongay, Wladek Walukiewicz, Jianwei Miao, Oscar Dubon, Junqiao Wu

**Affiliations:** 1 Department of Materials Science and Engineering, University of California, Berkeley, CA 94720 USA; 2 Materials Sciences Division, Lawrence Berkeley National Laboratory, Berkeley, CA 94720 USA; 3 Department of Physics & Astronomy and California NanoSystems Institute, University of California, Los Angeles, CA USA; 4 Beijing Computational Science Research Center, Beijing, China; 5 School of Aerospace Engineering, Beijing Institute of Technology, Beijing, China; 6 School for Engineering of Matter, Transport, and Energy, Arizona State University, Tempe, AZ 85287 USA

**Keywords:** Materials science, Two-dimensional materials

Correction to: *Nature Communications* 10.1038/s41467-020-19247-1, published online 23 October 2020.

The original version of this Article omitted the following from the Acknowledgements:

This research was partially conducted at the Center for Nanophase Materials Sciences, which is a DOE Office of Science User Facility.

This has now been corrected in both the PDF and HTML versions of the Article.

