# Supplementary material for: Chemical trends of deep levels in van der Waals semiconductors
Source: Nat Commun. 2020 Oct 23;11:5373. doi: 10.1038/s41467-020-19247-1 (PMC7584584; doi:10.1038/s41467-020-19247-1)
Supplement: Supplementary file 2 — Supplementary Information [file 41467_2020_19247_MOESM2_ESM.pdf]

# Supplementary Information

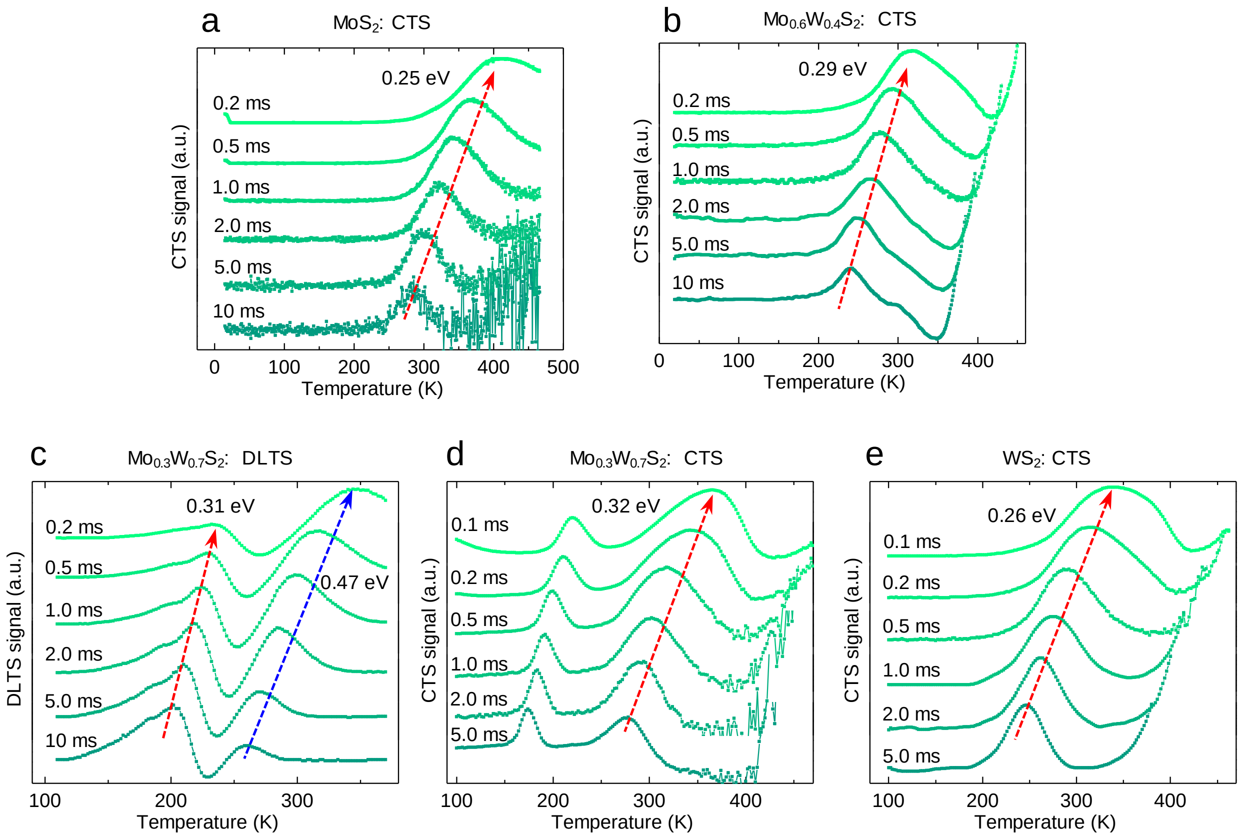


**Supplementary Figure 1: DLTS and CTS data for $Mo_{1-x}W_xS_2$ crystals.** The CTS spectra shows only the $V_S$ feature but with asymmetrical profile, in particular for curves with the rate window below 1.0 ms. The asymmetric shoulder at higher temperatures in the CTS spectra suggests a deeper energy level too weak to be analyzed, and is possibly caused by the DX center. The CTS spectrum of $Mo_{0.3}W_{0.7}S_2$ in Supplementary Fig. 1d shows an additional feature at lower temperatures than that with the activation energy of 0.32 eV. Its origin is currently unknown.

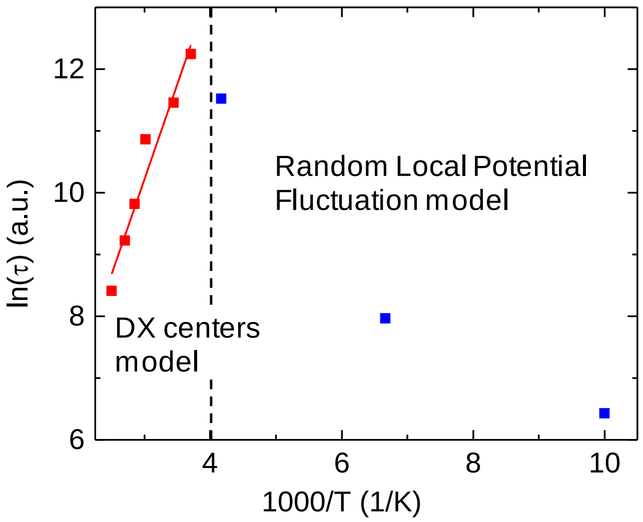


**Supplementary Figure 2: Arrhenius plot of PPC time constant for $MoS_2$ in the broad temperature range.** The DX center model explains the expeditious decay of PPC in the high-temperature regime (T > 270 K). When T < 240 K, the time constant of $MoS_2$ instead drops with decrease in temperature, which is explained by random local potential fluctuation (RLPF), a model that was proposed in previous reports[1,2]. In the RLPF model, random low-potential sites in the conduction band are spatially separated from those in the valence band, so photo-generated electrons (holes) tend to be trapped by these localized sites in the conduction (valence) band, which results in a long carrier lifetime and the PPC effect. These local potential fluctuation in the vdW materials could arise from disordered, charged native defects[2], or randomly distributed trapped charges on the $SiO_2$ substrate[3]. In details, at low temperatures (below ~100 K), photo-excited carriers are confined into these local sites and only contribute weakly to the current flow by hopping transport, hence leading to negligible PPC. As the temperature increases, more electrons gain sufficient kinetic energy to transfer from the localized states to delocalized states, forming a percolation network and thus contributing more to the conductivity, so the PPC effect becomes stronger and decays more slowly [1,4]. Note that when the thermal energy is sufficiently high, it excites all localized electrons from the local potential sites, consequently the PPC effect tends to saturate, and its time constant becomes fixed or only weakly depend on temperature[1].

Indeed, the PPC effect can arise from more than one mechanism. For example, in ZnCdSe[4], in the temperature range from 70 K to 220 K, the time constant of PPC rises with temperature because of the RLPF effect; but when T > 220 K, the time constant shows an opposite temperature dependence: the PPC decays faster as temperature grows. Both DX center and RLPF can cause the PPC effect but with distinct temperature dependencies. The energy barrier, $E_c$, of DX centers prevents photogenerated electrons from transferring to the localized DX centers, hence high temperature expedites the decay of the PPC. In contrast, in the RLPF mechanism, the local potential sites in the conduction band trap electrons that are frozen-out at low temperatures, thus, unlike DX centers, RLPF causes a faster decay of the PPC effect at lower temperatures.

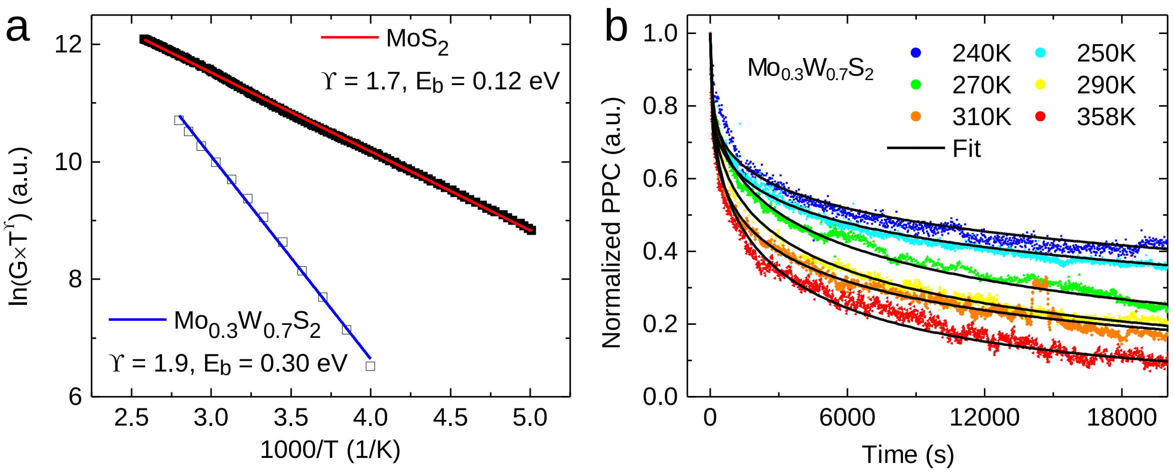


**Supplementary Figure 3**: **Temperature-dependent conductivity and photoconductivity. a**, Arrhenius plots of dark electron density ($n \propto G \times T^{\gamma}$) for $MoS_2$ and $Mo_{0.3}W_{0.7}S_2$ to determine binding energy $E_b$. **b**, Transient normalized-PPC curves at various temperatures for $Mo_{0.3}W_{0.7}S_2$, where the time constant is extracted by fitting with the stretched-exponential equation and shown in Fig. 3b.

We extract the thermal activation energy ($E_b$) for deep levels in $MoS_2$ and $Mo_{0.3}W_{0.7}S_2$ from the Arrhenius plot of the dark conductance versus inverse temperature as in Supplementary Fig. 3a. The carrier density ($n$) depends exponentially on temperature, $n \sim \exp(-E_b/k_BT)$, considering the Boltzmann distribution and the "full-slope" regime in the freeze-out curves of semiconductors, where only a small portion of the deep levels are ionized [5,6]. On the other hand, conductivity can be expressed by the Drude model as $\sigma = n\ e\ \mu$, where $\mu$ is the mobility following a temperature dependence of $\mu \sim T^{-\gamma}$ above ~ 200 K as reported in previous studies[7-9]. Combining these equations, $n$ is related to the conductance G and expressed as,

$$G \times T^{\gamma} \propto n \propto \exp\left(-\frac{E_b}{k_BT}\right). \qquad (1)$$

Arrhenius plot of Supplementary Eq. (1) yields an activation energy of 0.12 eV for $MoS_2$ by using an exponent of $\gamma$ = 1.7 as reported in literature[8]. We also found that the obtained value of $E_b$ is insensitive to the value of $\gamma$ used in the fitting, and only changes from 0.09 to 0.13 eV when $\gamma$ is changed from 0.5 to 2.5. Similarly, we extract the activation energy of 0.30eV for $Mo_{0.3}W_{0.7}S_2$ in Supplementary Fig. 3a, where $\gamma$ uses the interpolated value of 1.9 following the known values of 1.7 for $MoS_2$ and 2.0 for $WS_2$[8,9].

These $E_b$ values (0.12 eV for $MoS_2$ and 0.3 eV for $Mo_{0.3}W_{0.7}S_2$) are shallower than the energy level $E_i$ for sulfur vacancy ($V_S$) measured from $E_{CB}$. Therefore, they must originate from a defect level other than the $V_S$. As the only other deep level identified from DLTS is the DX centers, we assign the extracted $E_b$ to the energy distance between $E_{CB}$ and the $E_{DX}$. That is, $E_b = E_{CB} - E_{DX} = E_e - E_c$.

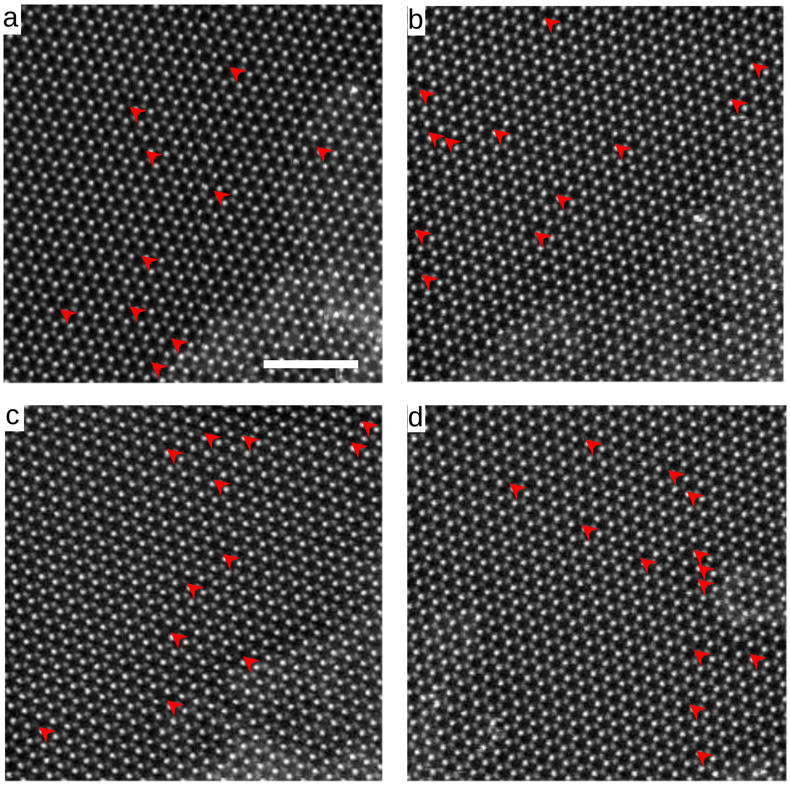


**Supplementary Figure 4: Imaging $V_S$ in monolayer $MoS_2$ with STEM.** Scale bar, 2nm. $V_S$ density is ~ 0.2 $nm^{-2}$ in our exfoliated monolayer $MoS_2$, corresponding to ~ $3\times10^{20}$ $cm^{-3}$ in multilayers. Subtracting the S vacancies induced by the electron beam yields the native density of > $1\times10^{20}$ $cm^{-3}$ (see methods in the main text), in agreement with literature[10,11].

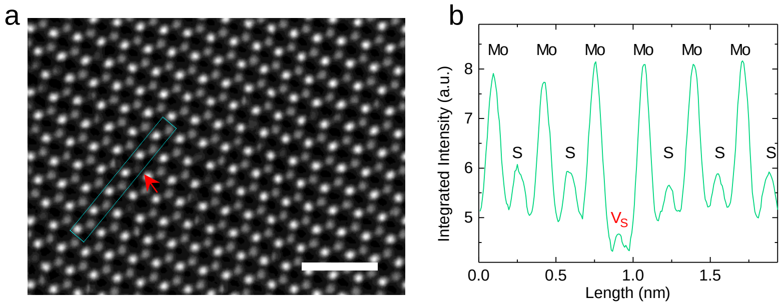


**Supplementary Figure 5: Intensity profiles of $V_S$ in b, corresponding to the boxed region in a.** Scale bar, 1 nm.

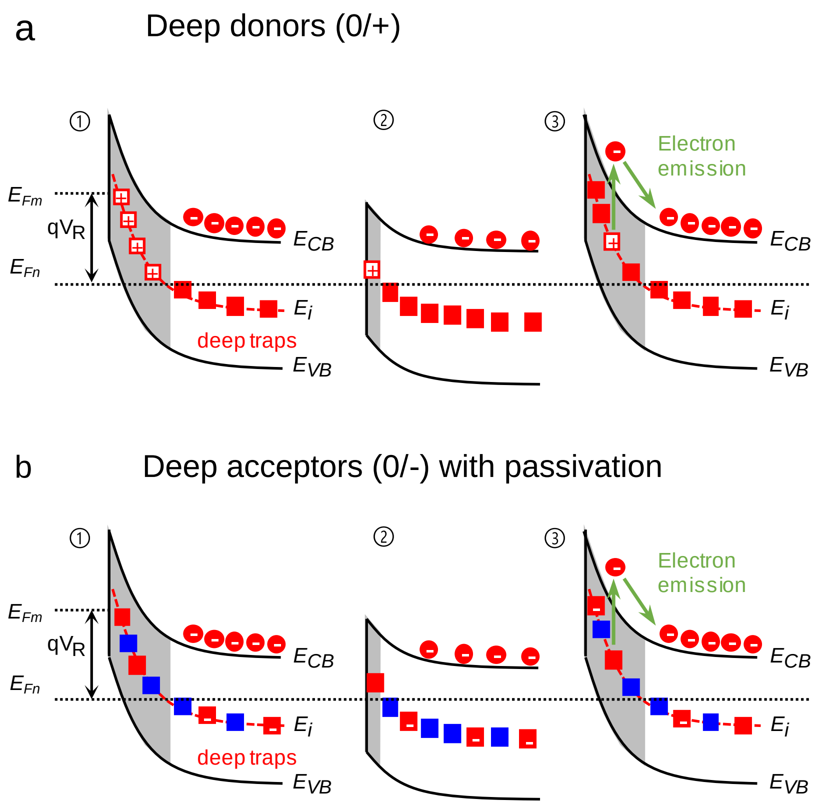


**Supplementary Figure 6: Band bending of a n-type Schottky junction in response to the biased voltage for deep donors in a, and partly passivated deep acceptors in b.** Blue blocks represent passivated (hence inactive and always neutral) deep acceptors. Stage ① - ③ correspond to those in Fig. 1c.

The concentration of $V_S$ determined from STEM in Supplementary Fig. 4 is about $1\times10^{20}$ $cm^{-3}$ in $MoS_2$, which is much higher than the free electron density of not intentionally doped $MoS_2$ on the order of ~ $10^{18}$ $cm^{-3}$ as reported in literature[7,12].This could be attributed to either compensation or passivation of the deep levels, as widely observed and reported in many traditional semiconductors[13-15]. Supplementary Figure 6a presents the band bending of deep donors with full occupancy in the ground state, akin to the schematic in the main text. However, deep traps in semiconductors may be passivated, and hence de-activated at equilibrium, as shown in the case of deep acceptors in Supplementary Fig. 6b, leading to only a small portion of traps in the depletion zone being active and able to emit electrons under the reverse bias. The mechanism of passivation of the deep levels is currently unknown in $MoS_2$, and is beyond the scope of this study, but the small capture cross section of $V_S$ deep acceptors and their weak attraction to free electrons may play a role.

**Supplementary Note 1: Calculation of deep level capture cross section from DLTS.**

Rewriting Eq. (1) yields $ln\left(\frac{T^2}{e_n}\right) = ln\left(\frac{1}{K\cdot\sigma_n}\right) + \left(\frac{E_{CB}-E_i}{k}\right)\frac{1000}{T}$, where the extrapolation in the Arrhenius plot (Fig. 2b) allows extraction of the capture cross section, $\sigma_n$. The constant $K$ is expressed as[5,16]

$$K = 2\left(\frac{2\pi m_e^* k}{h^2}\right)^{3/2}\left(\frac{3k}{m_{tc}^*}\right)^{1/2} = 3.26\times10^{21}\left[\frac{1}{cm^2K^2s}\right]\times\left(\frac{{m_e^*}^3}{m_{tc}^*}\right)^{1/2}, \qquad (2)$$

where $m_{tc}^*$ is the normalized thermal velocity effective mass, and $m_e^*$ is the normalized density of states mass. The latter mass has been determined to be 0.50 (normalized to the free electron mass) as reported by previous studies[17]. The former mass is expressed as[18]

$$m_{tc}^* = \frac{4m_l}{\left[1+\sqrt{m_l/m_t}\,\sin^{-1}(\delta)/\delta^2\right]^2}, \qquad (3)$$

where $\delta = \sqrt{(m_l - m_t)/m_l}$, and $m_l$ and $m_t$ are the longitudinal and transverse effective masses in the ellipsoidal energy surface[18]. Our DFT calculation determines $m_l$ and $m_t$ to be 0.62 and 0.55, respectively, hence giving $m_{tc}^*$ = 0.57. Finally, the capture cross section of $V_S$ is calculated to be ~$3.6\times10^{-18}$ $cm^2$ in $MoS_2$.

**Supplementary Note 2: Impact of our results: prediction of deep levels of anion impurities.**

The knowledge of $V_S$ attained in this study can be used to understand and predict energy levels of anion-substitutional impurities such as oxygen in $MoS_2$ or $WS_2$. In order to explain this prediction, we start with discussing the bonding / antibonding model for a di-atomic system with the secular equation[19,20]

$$\begin{vmatrix} E - E_{\mathrm{A0}} & V \\ V & E - E_{\mathrm{B0}} \end{vmatrix} = 0, \qquad (4)$$

where $E_{A0}$ and $E_{B0}$ (lower than $E_{A0}$) are the atomic levels, and $V$ is the interaction between $E_{A0}$ and $E_{B0}$ arising from the wavefunction overlap. Solving this equation yields two eigenvalues, corresponding to the molecular bonding and antibonding energy levels:

$$E_A = \frac{E_{A0}+E_{B0}}{2} + \frac{1}{2}\sqrt{(E_{A0} - E_{B0})^2 + 4V^2}, \qquad (5)$$

and

$$E_B = \frac{E_{A0}+E_{B0}}{2} - \frac{1}{2}\sqrt{(E_{A0} - E_{B0})^2 + 4V^2}. \qquad (6)$$

Then rewriting of Supplementary Eq. (4) gives

$$\Delta' = \frac{1}{2}\left(\sqrt{\Delta_0^2 + 4V^2} - \Delta_0\right), \qquad (7)$$

where $\Delta_0 = E_{A0} - E_{B0} > 0$ represents the energy difference between the initial atomic levels, and $\Delta' = E_A - E_{A0} > 0$ is the difference in energy between the atomic level and its originated molecular orbital (Supplementary Fig. 7). In order to determine the evolution of Supplementary Eq. (7) with the change in $\Delta_0$, the first-order differentiation is calculated as

$$\frac{d\Delta'}{d\Delta_0} = \frac{1}{2}\left(\frac{\Delta_0}{\sqrt{{\Delta_0}^2+4V^2}} - 1\right) < 0, \qquad (8)$$

indicating a monotonically decreasing function of Supplementary Eq. (7). This suggests that increase in the difference of the initial atomic levels will reduce the splitting between the atomic and molecular levels ($\Delta'$, see Supplementary Fig. 7).

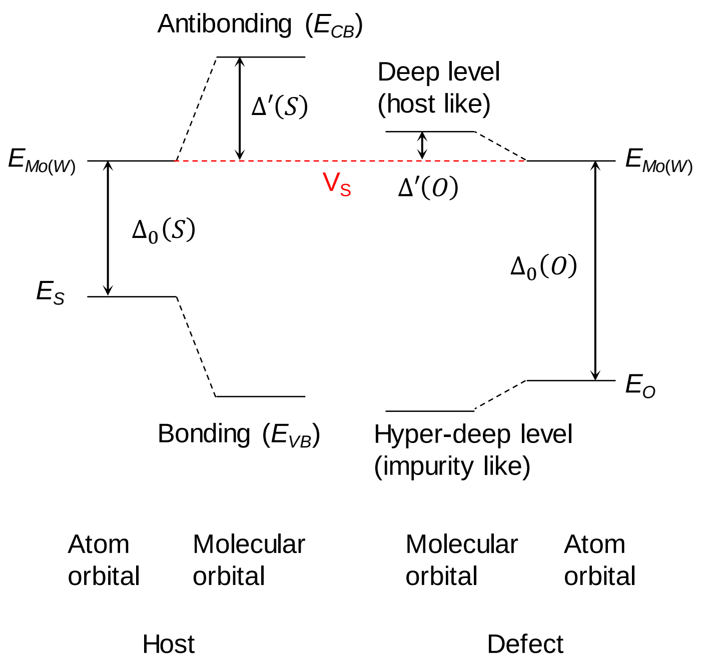


**Supplementary Figure 7: Bonding / antibonding model explaining the formation of deep levels in the bandgap of $MoWS_2$.** The left-hand side is schematic of the atomic levels of Mo(W) and S atoms and the resultant antibonding and bonding states in $MoS_2$ ($WS_2$). The right-hand side shows atomic level of the Mo(W) atom and substitutional O atom (defect) and the resultant deep and hyper-deep levels in $MoS_2$ ($WS_2$) with O defects.

Next, we discuss the formation of the energy level induced by more electronegative, anion substitutional impurities in $MoS_2$ or $WS_2$ in Supplementary Fig. 7. The left-hand side presents the creation of conduction and valence bands in $MoS_2$ following the simplest possible bonding / antibonding model. We note that, according to the origins of conduction band maximum (CBM) and the valence band minimum (VBM) in $MoS_2$ or $WS_2$[21], the antibonding state ($E_{CB}$) in Supplementary Fig. 7 can be defined as the CBM, while the bonding state ($E_{VB}$) is deeper than the VBM, so the difference between $E_{CB}$ and $E_{VB}$ is not equal to the bandgap. The energy difference between the atomic level of Mo(W) atom and the formed conduction band of Mo(W)$S_2$, $\Delta'(S)$, can be expressed by Supplementary Eq. (7). As calculated by DFT in Fig. 2d, the wavefunction of $V_S$ is composed mainly of orbitals of Mo(W) atoms, hence it is reasonable to assume that the position of $V_S$ level lies very close to the atomic level of Mo(W), $E_{Mo(W)}$, in Supplementary Fig. 7. Considering anion impurities such as oxygen substituting S in $MoWS_2$, the interaction between the O atom and its neighboring Mo(W) atoms forms two molecular levels, a deep level and a so-called hyper-deep level[22]. The latter is below the valence band and electrically inactive; in contrast, the former lies inside the bandgap and its wavefunctions is dominated by that of Mo(W), so it is called host-like defect level as shown in the right-hand side of Supplementary Fig. 7, akin to the nitrogen defect in GaP[22,23].

The low-lying oxygen atomic level with respect to the vacuum level means a more significant difference in the original energies ($\Delta_0(\mathrm{O}) = E_{Mo} - E_O$) than that in host materials ($\Delta_0(\mathrm{S}) = E_{Mo} - E_S$), resulting in the smaller splitting $\Delta'(\mathrm{O})$ in Supplementary Fig. 7, following the Supplementary Eq. (7) and (8). Due to the high electronegativity of O atom, the Mo(W)-O can form a more ionic bond with weaker wavefunction overlap and hence a smaller value of $V$ (Supplementary Eq. (7)),

compared to the more covalent Mo(W)-S bond. In summary, it is reasonable to predict that anion impurities would create deep levels with similar energies as the $V_S$, about 0.3 eV below the CBM, in $Mo_{1-x}W_xS_2$ of all compositions.

Finally, the analysis above is not limited to Mo(W) disulfides; all other transition metal chalcogenides may be similarly discussed in the context of native defect energies once the anion vacancy level is measured.

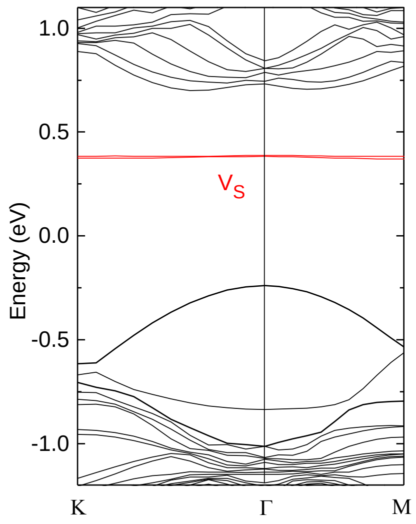


**Supplementary Figure 8: Band structure of multilayer $MoS_2$ with sulfur vacancies by DFT calculations, where $V_S$ indicates the energy level of sulfur vacancies.**

**Supplementary Note 3: Characterization of vdW crystals Field-effect transistors.**

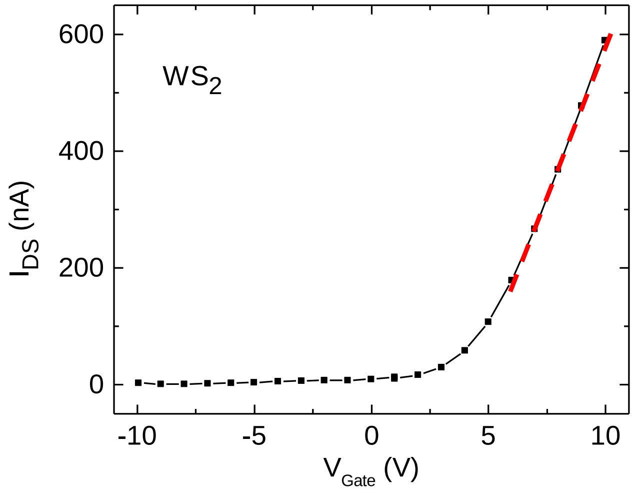


**Supplementary Figure 9: Source-drain current ($I_{DS}$) in response to the gate voltage ($V_{Gate}$) of field-effect transistors (FET) made of $WS_2$ at room temperature.**

It is known that undoped $MoS_2$ has a native electron density over orders of magnitude higher than in $WS_2$[24,25]. Here we explain it using the chemical trend of DX centers in these materials.

According to the data in Fig. 5b, we can extract the low-field field-effect mobility to be ~ 16 $cm^2/(V \cdot S)$ for $MoS_2$. Thus, the free carrier density ($n = 1/(e \cdot \mu \cdot \rho)$) of $MoS_2$ is calculated to be ~ $4\times10^{17}$ $cm^{-3}$, consistent with results in previous studies[12,24]. Similarly, the free electron concentration of $WS_2$ is determined to be $7\times10^{13}$ $cm^{-3}$ by the FET results in Supplementary Fig. 9, also in good agreement with literature[25].

Next, we discuss the effect of DX centers on the free carrier density in $MoS_2$ and $WS_2$. We assume the native donor density is on the same level in these two materials, but they are compensated to different extents by the DX centers as deep traps, because of their different energy depths in the bandgap of the hosts. Supplementary Equation 1 is then used to estimate the carrier density ratio of $MoS_2$ to $WS_2$

$$\frac{n(MoS_2)}{n(WS_2)} = exp\left(\frac{E_b(WS_2)-E_b(MoS_2)}{k_BT}\right), \qquad (9)$$

where $E_b(MoS_2)$ = 0.12 eV is the energy depth of DX centers in $MoS_2$, $E_b(WS_2)$ can be found by extrapolation to be ~ 0.38 eV based on the value of $MoS_2$ and $Mo_{0.3}W_{0.7}S_2$ in Fig.4c, and $k_BT$ = 26 meV at room temperature. Therefore, we obtain free carrier density ratio ($n(MoS_2)/n(WS_2)$) to be ~ $2\times10^4$ due to the charge compensation by DX centers. This is on the same order of magnitude as the value ($5\times10^3$) determined from FET measurements.

**Supplementary Note 4: Stray capacitance analysis and capacitance-voltage characterization.**

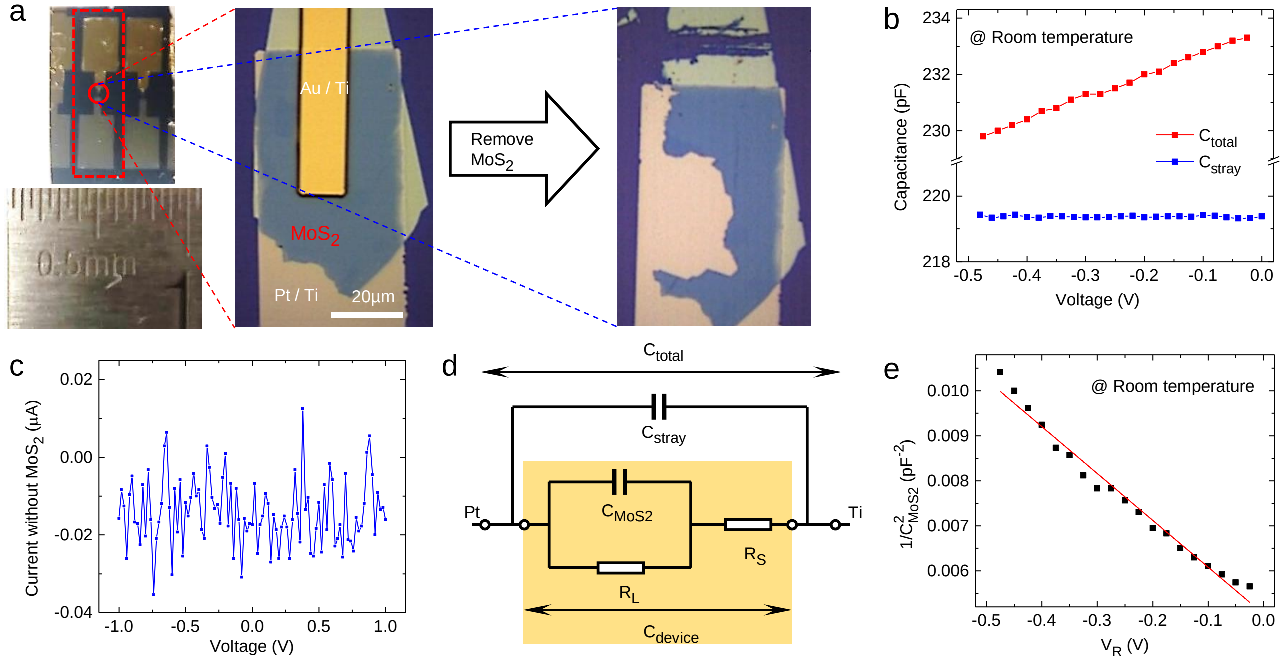


**Supplementary Figure 10: Stray capacitance analysis and capacitance-voltage characterization. a.** Optical image of an empty DLTS device for determination of parasitic capacitance by removing the flake of vdW material with a needle. The red circle represents the stacked parallel capacitor region with / without the flake of vdW material. **b**. Total capacitance ($C_{total}$, with flake) and stray capacitance ($C_{stray}$, removing flake) as a function of reverse bias at room temperature for the device in Supplementary Fig. 10a. **c**. Leakage current of the empty device in response to the bias voltage. **d.** Equivalent circuit of the device in Fig. 1a and Supplementary Fig. 10a. The yellow region represents the Schottky barrier circuit, showing a depletion capacitance of $MoS_2$ ($C_{MoS2}$) with a parallel leakage resistance ($R_L$) and series resistance of the non-depleted region ($R_S$). **e**. $1/C^2_{MoS2}$ *vs.* reverse voltage to characterize the dopant concentration for the device in Supplementary Fig. 10a at room temperature.

In order to fit the DLTS instrument, the electrodes of devices were designed to be on the size of several millimeters (Supplementary Fig. 10a), but the sample area is about tens of micrometers (Fig. 1a), so the large electrodes inevitably introduce parasitic coplanar capacitance, called stray capacitance, (Supplementary Fig. 10 b and d), whose magnitude is difficult to theoretically estimate due to the irregular geometry. This stray capacitance, even with a large value, is understandably insensitive to the external bias voltage, thereby not affecting the DLTS signal, because the DLTS records the differential capacitance within a rate window under reverse bias (Fig. 1c). Meanwhile, the weak, constant leakage current (~ 0.02 µA, Supplementary Fig. 10c) in the empty device (Supplementary Fig. 10a) ensures the accuracy and reliability of the CTS measurements in Supplementary Fig. 1.

We can determine the $MoS_2$ capacitance by measuring the difference between total capacitance ($C_{total}$, with sample) and stray capacitance ($C_{stray}$, removing sample) in Supplementary Fig. 10 a and b, so the curve of $1/C^2_{MoS2}$ vs. $V_R$ in Supplementary Fig. 10e allows us to obtain the built-in potential

($\Phi_{bi}$) of ~ 0.5 V for $MoS_2$/Pt Schottky diode and the dopant concentration of ~ $3\times10^{18}$ $cm^{-3}$ in nominally not intentionally doped $MoS_2$ ($N_d$) at room temperature, via its intercept and the slope according to[26]

$$\frac{1}{C_{MoS2}^2} = \frac{2(|\Phi_{bi}|+|V_R|)}{qN_d\varepsilon_r\varepsilon_0A^2}, \qquad (10)$$

where $\varepsilon_r$ = 11 is the dielectric constant of multilayer $MoS_2$[27], $V_R$ is the reverse biased voltage (Fig. 1c), $A$ is the area of the depletion zone, and $C_{MoS2}$ is close to $C_{device}$ (= $C_{total}$ - $C_{stray}$). These two extracted parameters for $MoS_2$ are consistent with previous results in literature[7,12,28]. As a result, the depletion width of the Schottky junction can be expressed as[26]

$$W = \sqrt{\frac{2\varepsilon_r\varepsilon_0(|\Phi_{bi}|+|V_R|)}{qN_d}}, \qquad (11)$$

and estimated to be ~ 22 nm under the reverse bias of 0.5 V at room temperature. Note that the dopant concentration determined here is significantly higher than the free electron density measured by FET in Supplementary Fig. 9, which is attributed to the trapping of free electrons by DX centers.

The linear $1/C^2$ *vs.* $V_R$ curve indicates a roughly uniform distribution of dopants and nearly step junction profile of space charge density near the surface of the $MoS_2$/Pt Schottky diode[16,29], hence confirming the feasibility of Supplementary Eq. (11) to extract the depletion width.

Although it is reasonable to assume a nearly step junction profile for the space charge, in reality the free carrier density varies exponentially within the depletion zone, so a Debye screening length (or Debye tail, Debye incursion) is defined to express the abruptness of the space charge distribution near the edge of the depletion zone, which can be written as[16]

$$L_D = \sqrt{\frac{\varepsilon_r\varepsilon_0kT}{q^2N_d}}. \qquad (12)$$

In our case, the high dopant concentration ($N_d$ ~ $3\times10^{18}$ $cm^{-3}$) yields a Debye length of ~ 2 nm at room temperature, which is on the same order with that in heavily doped silicon[16]. The depletion width (~22 nm) is more than ten times greater than this Debye length, which in turn justifies the sharp, nearly step - function profile of space charge[16].

In the above description, we assume that $C_{MoS2}$ is almost equal to $C_{device}$ by omitting the effect of the leakage resistance ($R_L$) and the series resistance ($R_S$). When subtracting the parallel stray capacitance, the measured capacitance, $C_{device}$ in the circuit within the yellow shadow (Supplementary Fig. 10d), is related to $C_{MoS2}$ by[16]

$$\frac{C_{MoS2}}{C_{device}} = \left(1+\frac{R_S}{R_L}\right)^2 + \left(\frac{R_S}{1/\omega C_{MoS2}}\right)^2, \qquad (13)$$

where $\omega$ is the frequency of a.c. voltage during the capacitance measurement and is 1 MHz in our case. Accurate test of the depletion capacitance and hence the depletion width requires that $R_S << R_L$ and $R_S << 1/\omega C_{MoS2}$, such that the capacitive impedance, $C_{MoS2}$, dominates the circuit element[16,29,30]. The leakage resistance ($R_L$) and series resistance ($R_S$) can be approximately estimated from the reverse and forward bias current of the Schottky junction to be 80 kΩ and 2.5 kΩ under the reverse bias of 0.2V at 320 K (Fig. 1e), meeting the requirement of $R_S << R_L$. Given that $C_{MoS2} \approx C_{total} - C_{stray}$, $1/\omega C_{MoS2}$ = 77 kΩ >> $R_S$ at 320 K. Therefore, Supplementary Equation (13) gives $C_{MoS2}$ ~ $C_{device}$, which justifies the reliability of the capacitance measurements. We note that the large leakage

current under reverse bias, called ‘soft’ reverse characteristics, may be attributed to the tunneling effect or the lowering of Schottky barrier height by image forces, as commonly reported in the Schottky junctions formed by low dimensional materials[31-33].

**Supplementary Note 5: Thermodynamic interpretation of Arrhenius plots in DLTS.**

The defect energy level in semiconductors is defined as the change of chemical potential due to the formation of a pair of charged carrier and ionized defect[34,35]. The chemical potential thermodynamically means the variation of Gibbs free energy during the capture or emission of an electron at constant pressure and temperature. Thus, based on these definitions, the Arrhenius equation of the thermal emission rate in Eq. (1) can be rewritten as[35]

$$\frac{e_n}{T^2} = K\sigma_n \exp\left(-\frac{\Delta \mathrm{G}(T)}{k_B T}\right), \qquad (14)$$

where $\Delta\mathrm{G}(T) = |E_{CB} - E_i|$ and is the activation energy for electron emission from the deep state to the conduction band edge. At the same time, the Gibbs free energy is defined by the thermodynamic identity as $\Delta\mathrm{G}(T) = \Delta\mathrm{H} - \mathrm{T}\Delta\mathrm{S}$, where ΔH and ΔS represent the changes in enthalpy and entropy, respectively. Therefore, Supplementary Equation (14) becomes[16]

$$\frac{e_n}{T^2} = K[\exp\left(\frac{\Delta \mathrm{S}}{k_B}\right)\sigma_n] \exp\left(-\frac{\Delta \mathrm{H}}{k_B T}\right), \qquad (15)$$

and hence the slope of the Arrhenius plot via Eq. (1) yields an average of enthalpy change over the temperature range of this plot, considering the generally weak temperature dependence of ΔH[16]. The difference between ΔG and ΔH mainly arises from the lattice vibrational contribution to ΔS due to the coupling of occupied deep states to the lattice, and therefore, it is usually negligible when electrons are excited from the traps to conduction band without changing the bonding configuration (ΔS ~ 0)[36]. Thus, in this study, it is reasonable to consider the measured Arrhenius slope from DLTS as the activation energy for $V_S$ states, because our DFT calculations do not observe lattice relaxation or entropy change during the transfer of electrons between the $V_S$ defect and the conduction band edge.

With regard to DX centers, most of previous studies on group III-V semiconductors also neglected the difference between ΔG and ΔH[37-43], despite the occurrence of lattice relaxation when a DX center switches to the electron-donating state. In this study, we do not consider this difference for DX centers in vdW crystals. On the other hand, in order to obtain the exact activation energy, ΔG(T), via Supplementary Eq. (14), one needs to measure the values of both $e_n$ and $\sigma_n$ at desired temperatures. The emission rate, $e_n$, can be determined by the DLTS or transient capacitance test, while the capture cross section is usually measured using the diode short-circuiting technique[44,45], which is out of the scope of this study.

The main text and Supplementary Note 1 show the extraction of capture cross section, $\sigma_n$, of $V_S$ deep state via the intercept of the Arrhenius plot in Fig. 2b. However, we note that, based on Supplementary Eq. (15), this intercept more accurately represents the product $\exp\left(\frac{\Delta \mathrm{S}}{k_B}\right)\sigma_n$, rather than just $\sigma_n$. Experimentally, one could measure the latter using the diode shorting-circuiting technique[44,45]to eventually determine the prefactor, ΔS, by temperature-dependent Gibbs free energy (ΔG = ΔH - TΔS).

**Supplementary Note 6: Activation energy of DX centers by Arrhenius plot of DLTS.**

Unlike regular deep levels which have no capture/emission barriers, such as the $V_S$ state, for DX centers the Arrhenius plot of the DLTS spectrum extracts the emission barrier $E_e$, which is not the energy of the DX center directly measured from the conduction or valence band edges. This is because, in the case of DX centers, the energy barrier, $E_c$ in the configurational coordinate diagram (CCD, Fig. 3c and 4c), must be overcome in order for an electron to be trapped by defects, hence leading to a strongly temperature-dependent capture cross section[5],

$$\sigma_{n,DX} = \sigma_\infty \exp\left(-\frac{E_c}{k_B T}\right). \qquad (16)$$

Combining Supplementary Eq. (16) and Eq. (1) gives

$$\frac{e_n}{T^2} = K\sigma_\infty \exp\left(-\frac{|E_{CB}-E_i|+E_c}{k_B T}\right), \qquad (17)$$

where $|E_{CB} - E_i| + E_c$ is equal to the emission energy, $E_e$, in the CCD (Fig. 3c) without considering the entropy change, and $E_i$ is $E_{DX}$.

To sum up, the DLTS spectrum measures the activation energy or binding energy ($E_b$) for normal defects such as the $V_S$ states, while for DX centers, DLTS yields the emission energy ($E_e$), the summation of binding energy ($E_b$) and capture barrier ($E_c$).

**Supplementary Note 7: Excluding a surface depletion mechanism for the PPC effect.**

Although a surface depletion model was used to explain PPC effects in some low-dimensional systems[46,47], this model is unlikely to explain our observed PPC in $MoS_2$ and alloys. This is because PPC effects induced by surface depletion, for instance, in Si NWs or α-$In_2Se_3$ nanosheets[46,47], originate from self-assembled molecules on the surface or oxygen ions adsorbed from the environment. However, our STEM images (Fig. 1b) confirm the absence of adsorbents or contamination on the surface. Moreover, all the PPC tests were completed in high vacuum (~ $10^{-6}$ torr) after annealing at 400 K in vacuum for at least one day to remove possible adsorbents. In the meantime, such surface depletion mechanism usually induces only a weak PPC with a short decay constant (*e.g.*, ~ seconds at room temperature)[46], in stark contrast to the long PPC decay time we observed (~ $10^5$ s for multilayer $MoS_2$ at room temperature). Therefore, our PPC effect observed in $MoS_2$ and alloys is unlikely to be caused by any surface modification of the samples.

## Supplementary References


1 Jiang, H. & Lin, J. Percolation transition of persistent photoconductivity in II-VI mixed crystals. *Physical review letters* **64**, 2547 (1990).

2 Wu, Y.-C. *et al.* Extrinsic origin of persistent photoconductivity in monolayer MoS 2 field effect transistors. *Scientific reports* **5**, 11472 (2015).

3 Xue, J. *et al.* Scanning tunnelling microscopy and spectroscopy of ultra-flat graphene on hexagonal boron nitride. *Nature materials* **10**, 282-285 (2011).

4 Jiang, H. & Lin, J. Persistent photoconductivity and related critical phenomena in Zn 0.3 Cd 0.7 Se. *Physical Review B* **40**, 10025 (1989).

5 McCluskey, M. D. & Haller, E. E. *Dopants and defects in semiconductors*. (CRC press, 2018).

6 Chand, N. *et al.* Comprehensive analysis of Si-doped Al x Ga 1− x As (x= 0 to 1): Theory and experiments. *Physical Review B* **30**, 4481 (1984).

7 Radisavljevic, B. & Kis, A. Mobility engineering and a metal–insulator transition in monolayer MoS 2. *Nature materials* **12**, 815-820 (2013).

8 Perera, M. M. *et al.* Improved carrier mobility in few-layer MoS2 field-effect transistors with ionic-liquid gating. *ACS nano* **7**, 4449-4458 (2013).

9 Xu, S. *et al.* Universal low-temperature Ohmic contacts for quantum transport in transition metal dichalcogenides. *2D Materials* **3**, 021007 (2016).

10 Hong, J. *et al.* Exploring atomic defects in molybdenum disulphide monolayers. *Nature communications* **6**, 1-8 (2015).

11 Qiu, H. *et al.* Hopping transport through defect-induced localized states in molybdenum disulphide. *Nature communications* **4**, 1-6 (2013).

12 Radisavljevic, B., Radenovic, A., Brivio, J., Giacometti, V. & Kis, A. Single-layer MoS 2 transistors. *Nature nanotechnology* **6**, 147 (2011).

13 Lagowski, J., Kaminska, M., Parsey Jr, J., Gatos, H. & Lichtensteiger, M. Passivation of the dominant deep level (EL2) in GaAs by hydrogen. *Applied Physics Letters* **41**, 1078-1080 (1982).

14 Dautremont‐Smith, W. *et al.* Passivation of deep level defects in molecular beam epitaxial GaAs by hydrogen plasma exposure. *Applied physics letters* **49**, 1098-1100 (1986).

15 Nabity, J. *et al.* Passivation of Si donors and DX centers in AlGaAs by hydrogen plasma exposure. *Applied physics letters* **50**, 921-923 (1987).

16 Blood, P. & Orton, J. W. *The electrical characterization of semiconductors: majority carriers and electron states*. Vol. 2 (Academic press London, 1992).

17 Peelaers, H. & Van de Walle, C. G. Effects of strain on band structure and effective masses in MoS 2. *Physical Review B* **86**, 241401 (2012).

18 Green, M. A. Intrinsic concentration, effective densities of states, and effective mass in silicon. *Journal of Applied Physics* **67**, 2944-2954 (1990).

19 Ci, P. *et al.* Quantifying van der Waals interactions in layered transition metal dichalcogenides from pressure-enhanced valence band splitting. *Nano letters* **17**, 4982-4988 (2017).

20 Burns, G. *Solid State Physics*. (Elsevier Science, 1985).

21 Kang, J., Tongay, S., Zhou, J., Li, J. & Wu, J. Band offsets and heterostructures of two-dimensional semiconductors. *Applied Physics Letters* **102**, 012111 (2013).

22 Hjalmarson, H. P., Vogl, P., Wolford, D. J. & Dow, J. D. Theory of substitutional deep traps in covalent semiconductors. *Physical Review Letters* **44**, 810 (1980).

23 Yu, P. Y. & Cardona, M. *Fundamentals of semiconductors: physics and materials properties*. (Springer, 1996).

24 Kwak, J. Y. *et al.* Electrical characteristics of multilayer MoS2 FET's with MoS2/graphene heterojunction contacts. *Nano letters* **14**, 4511-4516 (2014).

25 Braga, D., Gutiérrez Lezama, I., Berger, H. & Morpurgo, A. F. Quantitative determination of the band gap of WS2 with ambipolar ionic liquid-gated transistors. *Nano letters* **12**, 5218-5223 (2012).

26 Hu, C. *Modern semiconductor devices for integrated circuits*. Vol. 2 (Prentice Hall Upper Saddle River,

New Jersey, 2010).
27 Chen, X. *et al.* Probing the electron states and metal-insulator transition mechanisms in molybdenum disulphide vertical heterostructures. *Nature communications* **6**, 6088 (2015).
28 Kim, G.-S. *et al.* Schottky barrier height engineering for electrical contacts of multilayered MoS2 transistors with reduction of metal-induced gap states. *ACS nano* **12**, 6292-6300 (2018).
29 Pierret, R. F. *Semiconductor device fundamentals*. (Pearson Education India, 1996).
30 Goodman, A. M. Metal—Semiconductor barrier height measurement by the differential capacitance method—One carrier system. *Journal of Applied Physics* **34**, 329-338 (1963).
31 Kumar, S. *et al.* Influence of barrier inhomogeneities on transport properties of Pt/MoS2 Schottky barrier junction. *Journal of Alloys and Compounds* **797**, 582-588 (2019).
32 Rhoderick, E. H. & Rhoderick, E. *Metal-semiconductor contacts*. (Clarendon Press Oxford, 1978).
33 Chen, C.-C., Aykol, M., Chang, C.-C., Levi, A. & Cronin, S. B. Graphene-silicon Schottky diodes. *Nano letters* **11**, 1863-1867 (2011).
34 Van Vechten, J. & Thurmond, C. Entropy of ionization and temperature variation of ionization levels of defects in semiconductors. *Physical Review B* **14**, 3539 (1976).
35 Thurmond, C. The standard thermodynamic functions for the formation of electrons and holes in Ge, Si, GaAs, and GaP. *Journal of the Electrochemical Society* **122**, 1133 (1975).
36 Almbladh, C.-O. & Rees, G. Statistical mechanics of electronic energy levels in semiconductors. *Solid State Communications* **41**, 173-176 (1982).
37 Criado, J., Gomez, A., Munoz, E. & Calleja, E. Deep level transient spectroscopy signature analysis of DX centers in AlGaAs and GaAsP. *Applied physics letters* **49**, 1790-1792 (1986).
38 Calleja, E., Gomez, A., Munoz, E. & Camara, P. Fine structure of the alloy ‐ broadened thermal emission spectra from DX centers in GaAlAs. *Applied physics letters* **52**, 1877-1879 (1988).
39 Kasu, M., Fujita, S. & Sasaki, A. Observation and characterization of deep donor centers (DX centers) in Si ‐ doped AlAs. *Journal of applied physics* **66**, 3042-3046 (1989).
40 Calleja, E., Gomez, A. & Munoz, E. Direct evidence of the DX center link to the L ‐ conduction ‐ band minimum in GaAlAs. *Applied physics letters* **52**, 383-385 (1988).
41 Mooney, P., Theis, T. & Wright, S. Effect of local alloy disorder on emission kinetics of deep donors (DX centers) in Al x Ga1− x As of low Al content. *Applied physics letters* **53**, 2546-2548 (1988).
42 Kumagai, O., Kawai, H., Mori, Y. & Kaneko, K. Chemical trends in the activation energies of DX centers. *Applied physics letters* **45**, 1322-1323 (1984).
43 Theis, T., Mooney, P. & Wright, S. Electron Localization by a Metastable Donor Level in n− GaAs: A New Mechanism Limiting the Free-Carrier Density. *Physical review letters* **60**, 361 (1988).
44 Brotherton, S. & Lowther, J. Electron and hole capture at Au and Pt centers in silicon. *Physical Review Letters* **44**, 606 (1980).
45 Brotherton, S. & Bicknell, J. The electron capture cross section and energy level of the gold acceptor center in silicon. *Journal of Applied Physics* **49**, 667-671 (1978).
46 Anandan, M. *et al.* High-responsivity broad-band sensing and photoconduction mechanism in direct-Gap α-In2Se3 nanosheet photodetectors. *Nanotechnology* **31**, 465201 (2020).
47 Zhao, X., Tu, P., He, J., Zhu, H. & Dan, Y. Cryogenically probing the surface trap states of single nanowires passivated with self-assembled molecular monolayers. *Nanoscale* **10**, 82-86 (2018).
